# A case of severe osteomalacia caused by Tubulointerstitial nephritis with Fanconi syndrome in asymptomotic primary biliary cirrhosis

**DOI:** 10.1186/s12882-015-0184-4

**Published:** 2015-11-11

**Authors:** Shintaro Yamaguchi, Tatsuya Maruyama, Shu Wakino, Hirobumi Tokuyama, Akinori Hashiguchi, Shinichiro Tada, Koichiro Homma, Toshiaki Monkawa, James Thomas, Kazutoshi Miyashita, Isao Kurihara, Tadashi Yoshida, Konosuke Konishi, Koichi Hayashi, Matsuhiko Hayashi, Hiroshi Itoh

**Affiliations:** Department of Internal Medicine, Keio University School of Medicine, 35 Shinanomachi, Shinjuku-ku, Tokyo, 160-8582 Japan; Apheresis and Dialysis Center, Keio University School of Medicine, 35 Shinanomachi, Shinjuku-ku, Tokyo, 160-8582 Japan; Department of Pathology, Keio University School of Medicine, 35 Shinanomachi, Shinjuku-ku, Tokyo, 160-8582 Japan; Medical education Center, Keio University School of Medicine, 35 Shinanomachi, Shinjuku-ku, Tokyo, 160-8582 Japan; Center for Clinical Research, Keio University School of Medicine, 35 Shinanomachi, Shinjuku-ku, Tokyo, 160-8582 Japan

**Keywords:** Osteomalacia, Tubulointerstitial nephritis, Fanconi syndrome, Primary biliary cirrhosis, Mitochondrial cytopathy

## Abstract

**Background:**

Primary biliary cirrhosis (PBC) is an immune-mediated chronic cholestatic liver disease, characterized by increased concentrations of serum IgM and the presence of circulating anti-mitochondrial antibodies. Although bone diseases such as osteoporosis or osteodystrophy are commonly associated with PBC, osteomalacia which is caused by abnormal vitamin D metabolism, mineralization defects, and phosphate deficiency has not been recognized as a complication of PBC.

**Case presentation:**

We report the case of a 49-year-old Japanese woman who complained of multiple fractures. Hypophosphatemic osteomalacia was diagnosed from a low serum phosphorus level, 1,25-dihydroxyvitamin D_3_ level_,_ high levels of bone specific alkaline phosphatase and the findings of bone scintigraphy, although a bone biopsy was not performed. Twenty four hour urine demonstrated a low renal fractional tubular reabsorption of phosphate, increased fractional excretion of uric acid and generalized aminoaciduria. An intravenous bicarbonate loading test suggested the presence of proximal renal tubular acidosis (RTA). These biochemical data indicated Fanconi syndrome with proximal RTA. A kidney biopsy demonstrated the features of tubulointerstitial nephritis (TIN).

The patient was also suspected as having primary biliary cirrhosis (PBC) because of high levels of alkaline phosphatase, IgM and the presence of anti-mitochondrial M2 antibody, though biochemical liver function was normal. Sequential liver biopsy was compatible with PBC and the diagnosis of PBC was definite. After administration of 1,25 dihydroxyvitamin D_3,_ neutral potassium phosphate, sodium bicarbonate for osteomalacia and subsequent predonizolone for TIN, symptoms of fractures were relieved and renal function including Fanconi syndrome was ameliorated.

**Conclusion:**

In this case, asymptomatic PBC was shown to induce TIN with Fanconi syndrome with dysregulation of electrolytes and vitamin D metabolism, which in turn led to osteomalacia with multiple fractures. Osteomalacia has not been recognized as a result of the renal involvement of PBC. PBC and its rare complication of TIN with Fanconi syndrome should be considered in adult patients with unexplained osteomalacia even in the absence of liver dysfunction.

## Background

Primary biliary cirrhosis (PBC) is an immune-mediated chronic cholestatic liver disease, characterized by non-suppurative destruction of interlobular bile ducts [1, 2]. Serologically, PBC is accompanied by increased concentrations of serum immunoglobulin M (IgM) and the presence of circulating anti-mitochondrial antibodies [1]. Although bone diseases such as osteoporosis or osteodystrophy, are commonly associated with PBC, osteomalacia, which is caused by abnormal vitamin D metabolism, mineralization defects, and phosphate deficiency, has not been frequently complicated with PBC [3, 4]. In renal complication of PBC, distal renal tubular acidosis (RTA) has been reported with the prevalence rates of 30 to 60 % generally in asymptomatic or latent condition [5, 6]. Tubulointerstitial nephritis (TIN) and subsequent Fanconi syndrome, a type of proximal tubular defects, have been more rarely reported in patients with PBC [7–9].

We report herein a rare case of hypophosphatemic osteomalacia caused by TIN with Fanconi syndrome in asymptomatic PBC. Multiple fractures due to hypophosphatemic osteomalacia were almost ameliorated after the administration of 1,25 dihydroxyvitamin D_3,_ neutral potassium phosphate, and sodium bicarbonate. The renal function also remained stable after subsequent administration of middle-dose corticosteroids.

## Case presentation

**Fig. 1:**
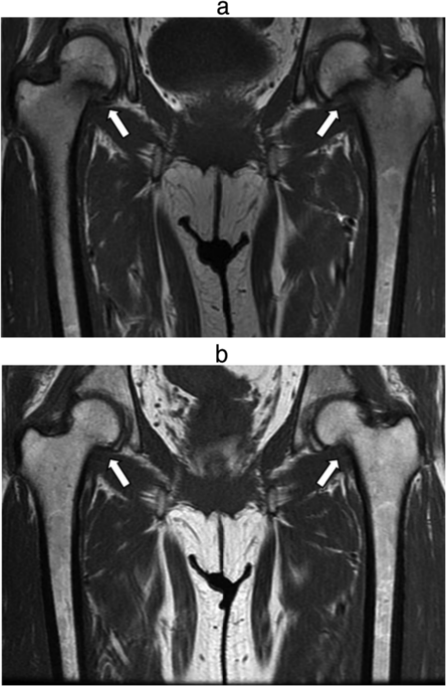
MRI findings of incomplete bilateral transcervical fractures (Arrows) before (**a**) and after (**b**) the treatment

**Fig. 2:**
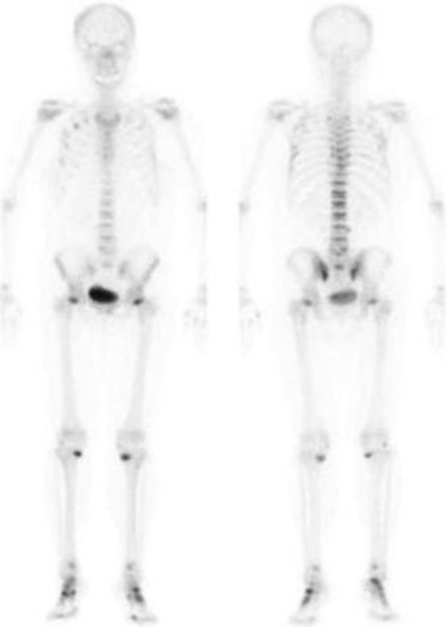
Bone scintigram showing multiple hot spots especially over multiple joints and ribs

In November 2007, a 49-year-old Japanese woman was referred to our hospital from an orthopaedist complaining of a one year history of sustained difficulty walking and severe bilateral hip pain. Her height was 155.5 cm and body weight was 61.0 kg. Laboratory data showed a creatinine of 1.4 mg/dl, potassium 2.8 mmol/l, calcium 9.5 mg/dl, phosphorus 2.5 mg/dl, uric acid 1.5 mg/dl, normoglycaemic glycosuria and metabolic acidemia (pH 7.30, HCO_3_^−^ 17.4 mmol/l) (Table 1). Bilateral transcervical fractures were confirmed by MRI (Fig. 1a) and bone scintigraphy showed multiple hot spots in her joints and ribs, compatible with osteomalacia (Fig. 2). Hypophosphatemic osteomalacia was diagnosed clinically from a low serum level of phosphorus, 1,25-dihydroxyvitamin D_3_ (11.0 pg/dl), high levels of bone specific alkaline phosphatase (67.5 IU/l), and the findings of bone scintigram, although a bone biopsy was not performed. Tumor-induced osteomalacia (TIO) was ruled out by total body survey with whole body computed tomography or endoscopic surveillances and a normal blood FGF23 (22.1 pg/ml, 10–50 pg/ml) level.

**Table 1:** Baseline data of the patient on admission in November 2007

Peripheral blood	Urinalysis
white blood cell count 6800/μl	pH 6.0
hemoglobin 13.5 g/dl	protein (3+)
platelet 20.1 × 10^4^/μl	urine sugar (4+)
Biochemistry	
total protein 8.7 g/dl,	bone specific alkaline phosphatase 67.5 IU/l
albumin 4.6 g/dl	γ-glutamyl transpeptidase 18 IU/l
total bilirubin 0.7 mg/dl	1,25-dihydroxyvitamine D 11.0 pg/dl
blood urea nitrogen 19.4 mg/dl	intact parathyroid hormone 46 pg/ml
creatinine 1.4 mg/dl	parathyroid hormone-related protein <1.1 pg/ml
uric acid 1.5 mg/dl	calcitonin 24 pg/ml
sodium 137.8 mEq/l	osteocalcin 7.6 ng/ml
potassium 2.8 mEq/l	fibroblast growth factor-23 22.1 pg/ml
chloride 108 mEq/l	IgG 1228 mg/dl
calcium 9.5 mg/dl	IgA 201 mg/dl
phosphorus 2.3 mg/dl	IgM 1084 mg/dl
fasting plasma glucose 98 mg/dl	anti-nuclear antibody 1:1280
hemoglobin A_1c_ 4.8 %	anti-SS-A/R_o_ antibody (−)
total cholesterol 249 mg/dl	anti-SS-B/La antibody (−)
c-reactive protein 0.17 mg/dl	anti-double stranded DNA antibody 3 IU/ml
lactate dehydrogenase 191 IU/l	Shirmer test (−)
aspartate aminotransferase 23 IU/l	serum M protein(−)
alanine aminotransferase 24 IU/l	urine Bence Jones Protein (−)
alkaline phosphatase 663 IU/l	anti-mitochondrial M_2_ antibody 10.0 index
Arterial blood gas analysis (Room Air)	24-h urine analysis
pH 7.306	Tmp/GFR 0.91
pO_2_ 89.0 torr	%TRP 37.9 %
pCO_2_ 35.9 torr	FEUA 43.9 %
HCO_3_^−^ 17.4 mmol/l	glucose 12.83 g
bicarbonate loading test	generalized aminoaciduria(+)
FEHCO_3_^−^ 13.25 %	protein 2.464 g
U-Bpco_2_ 50.1 mmHg	FEca 11.878 %,
	CrCl 45.76 ml/min/1.73 m^2^

Urinalysis showed pH 6.0 and twenty four hour urine demonstrated a low renal fractional tubular reabsorption of phosphate (Tmp/GFR 0.91), increased fractional excretion of uric acid (FEUA 43.9 %) and generalized aminoaciduria.

**Fig. 3:**
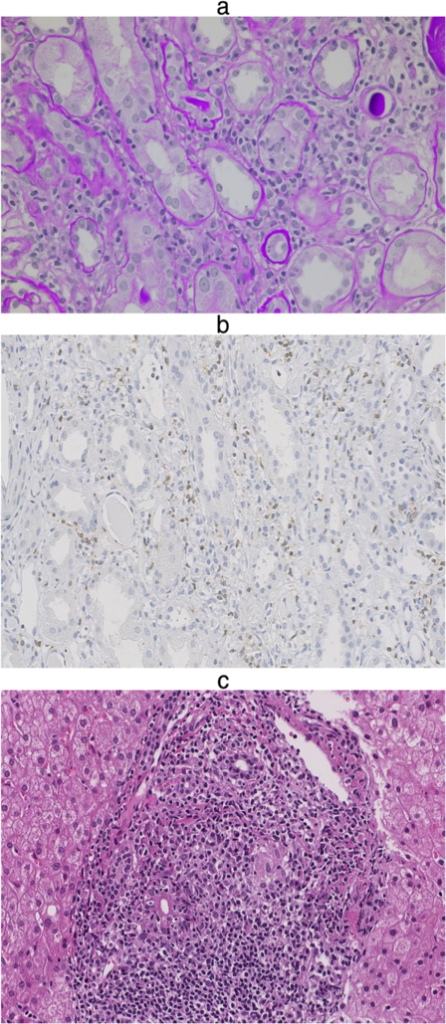
The Findings of Kidney and Liver Biopsies. **a**, **b** Kidney biopsy specimen; light microscopy shows (**a**) cellular infiltration in the proximal tubules and interstitium, and the presence of atrophic tubules (Hematoxylin-eosin staining) and (**b**) CD_3_^+^ cells infiltration in the renal interstitium. **c** Liver biopsy specimen; light microscopy shows the presence of a periportal cellular infiltrate

The calculated anion gap was 12.4 meq/l, with an inappropriately alkaline urine (pH 6.0), suggestive of distal type of renal tubular acidosis (RTA). An intravenous bicarbonate loading test to assess the tubular dysfunction demonstrated that the increased fractional excretion of bicarbonate (FEHCO_3_^−^ 13.25 %) and urine-blood (UB) Pco_2_ gradient (U-Bpco_2_ 50.1 mmHg), suggesting the presence of proximal type of RTA. NH_4_Cl loading test was not performed because of the presence of apparent metabolic acidemia. These biochemical data of blood and urine indicated Fanconi syndrome with proximal type of RTA. Tests for anti-SSA and anti-SSB were negative and serum angiotensin converting enzyme levels were normal. Furthermore, serum and urine immunoelectrophoresis revealed no monoclonal component. Any of the patient’s medication did not seem to be related to Fanconi syndrome. Light microscopic examination of a kidney biopsy demonstrated cellular infiltration of interstitium and proximal tubular epithelium composed mainly of CD_3_^+^ lymphocytes without glomerular involvement, consistent with features of TIN (Fig. 3a ,b).

The patient was also suspected to be diagnosed as PBC because of high levels of alkaline phosphatase (663 IU/l), high serum IgM levels (1084 mg/dl) and the presence of anti-mitochondrial M2 antibody (1:10). Sequential liver biopsy demonstrated the presence of a periportal cellular infiltrate (Fig. 3c), compatible with PBC though biochemical liver function was normal. The combination of these findings led to the patient’s diagnosis of osteomalacia caused by TIN with Fanconi syndrome in asymptomatic PBC.

The patient was started on therapy with calcitriol (1 μg/day), neutral potassium phosphate and sodium bicarbonate (6 g/day). She was subsequently administered middle-dose corticosteroid (20 mg/day of predonisolone) and ursodeoxycholic acid (300 mg/day). One year after the treatment, the patient became asymptomatic and renal function remained stable. Furthermore, MRI showed the bilateral transcervical fractures of the femoral neck had almost fully repaired (Fig. 1b). Treatment is currently being continued in the outpatient clinic.

## Discussion

We described a severe osteomalacia caused by TIN with Fanconi syndrome in asymptomatic PBC. Although osteomalcia is rarely observed in patients with PBC [4], malabsorption as a consequence of severe cholestasis and impaired ability to convert vitamin D to 25-hydroxivitamin D can cause osteomalacia in patients with advanced PBC [10, 11]. Our case was unique in that osteomalacia developed as a result of the rare renal involvement of asymptomatic PBC.

Osteomalacia is accelerated by inadequate renal phosphorus wasting and decreased uptake of active vitamin D as well as a decrease in 1α-hydroxylation of vitamin D [12, 13]. The serum concentration of 1,25 dihydroxyvitamin D_3_ which reflects the regulation of 1α-hydroxylation of vitamin D in the kidney is impaired in renal tubular diseases such as Fanconi syndrome [14, 15]. This patient had a low circulating level of 1,25 dihydroxyvitamin D_3_ which is presumed to be caused by Fanconi syndrome. Indeed, osteomalacia is a well-observed consequence of the Fanconi syndrome [16]. Fanconi syndrome also complicated with chronic hypophosphatemia and chronic acidemia, which considered to cause and aggravate osteomalacia [7, 17].

It is suggested that hepatic dysfunction is not implicated in the pathogenesis of TIN, while on the other hand, TIN in the present case may be pathogenically related to PBC [7]. First, lymphocytic infiltration is a pathological mutual feature of TIN and PBC. Autoreactive T lymphocytes of the tubular epithelium and interstitium may be driven by abnormal antigen expression such as mitochondrial antigen in hepatocytes and renal tubular cells [18]. T lymphocytes of the tubular epithelium are likely to be involved in the inhibition of proximal tubular function, leading to Fanconi syndrome. Second, Anti-M_2_ antibody in patients with PBC could interfere with intrarenal three mitochondrial enzymes: pyruvate dehydrogenase complex, branched-chain α-keto acid dehydrogenase complex and α-keto glutamate dehydrogenase complex [7, 9, 19]. Fanconi syndrome and TIN are typical renal features of mitochondrial cytopathies [20]. Although much remains to be elucidated about the pathogenesis of PBC and its extrahepatic complication of TIN with Fanconi syndrome, circulating anti-mitochondrial M_2_ antibody would induce PBC and TIN with Fanconi syndrome. Since the usual causes of Fanconi syndrome including metal intoxication were not confirmed, this patient was considered to have PBC-related TIN with Fanconi syndrome.

In summary, severe osteomalacia was considered to be caused by the rare renal involvement of asymptomatic PBC, which is TIN with Fanconi syndrome.

## Conclusions

Herein, the authors present a unique case of osteomalacia developed as a result of the rare renal involvement of asymptomatic PBC. As the prevalence of osteomalacia is increasing in aged societies where homebound elderly patients suffer from nutritional insufficiency, lack of sun exposure, medical histories of gastrointestinal surgery, and the possibility of malignancy, careful inspection for the cause of multiple fractures is necessary. We suggest that PBC and its rare complication of TIN with Fanconi syndrome should be considered in adult patients with unexplained osteomalacia even in the absence of liver function tests abnormalities.

## Consent

Written informed consent was obtained from the patient for publication of this case report and any accompanying images. A copy of the written consent is available for review by the Editor of this journal.

## Abbreviations

IgM: immunoglobulin M
PBC: Primary biliary cirrhosis
RTA: renal tubular acidosis
TIN: tubulointerstitial nephritis
TIO: Tumor-induced osteomalacia
